# The* uS8*,* uS4*,* eS31*, and* uL14* Ribosomal Protein Genes Are Dysregulated in Nasopharyngeal Carcinoma Cell Lines

**DOI:** 10.1155/2017/4876954

**Published:** 2017-07-16

**Authors:** Edmund Ui-Hang Sim, Kher-Lee Ng, Choon-Weng Lee, Kumaran Narayanan

**Affiliations:** ^1^Department of Molecular Biology, Faculty of Resource Science and Technology, Universiti Malaysia Sarawak, 94300 Kota Samarahan, Sarawak, Malaysia; ^2^Institute of Biological Sciences, University of Malaya, 50603 Kuala Lumpur, Malaysia; ^3^School of Science, Monash University, Bandar Sunway, 46150 Selangor, Malaysia; ^4^Department of Genetics and Genomics Sciences, Mount Sinai School of Medicine, New York, NY 10029, USA

## Abstract

The association of ribosomal proteins with carcinogenesis of nasopharyngeal carcinoma (NPC) has been established in a limited subset of ribosomal protein genes. To date, three ribosomal protein genes,* eL27 (L27)*,* eL41 (L41)*, and* eL43 (L37a)*, have been found to be differentially expressed in cell lines derived from NPC tumors. This raises the possibility of more ribosomal protein genes that could be associated with NPC. In this study, we investigated the expression profiles of eight ribosomal protein genes,* uS8 (S8), uS4 (S9), eS31 (S27a), eL6 (L6), eL18 (L18), uL14 (L23), eL24 (L24)*, and* eL30 (L30)*, in six NPC-derived cell lines (HONE-1, SUNE1, HK1, TW01, TW04, and C666-1). Their expression levels were compared with that of a nonmalignant nasopharyngeal epithelial cell line (NP69) using quantitative real-time PCR (RT-qPCR) assay. Of the eight genes studied, the expressions of four ribosomal protein genes* uS8 (S8), uS4 (S9), eS31 (S27a), *and* uL14 (L23) *were found to be significantly downregulated in NPC cell lines relative to NP69. Our findings provide novel empirical evidence of these four ribosomal protein genes as NPC-associated genetic factors and reinforce the relevance of ribosomal proteins in the carcinogenesis of nasopharyngeal cancer.

## 1. Introduction

Nasopharyngeal carcinoma (NPC) is a distinct malignant form of head and neck cancer that originates from the lateral or posterosuperior mucosal epithelium of the pharynx. The World Health Organization (WHO) classifies NPC as three major histopathologic types: Type I, keratinizing squamous cell carcinoma; Type IIa, nonkeratinizing differentiated cell carcinoma; and Type IIb, nonkeratinizing undifferentiated carcinoma. Type IIb is the most common and contributes to 60% and 95% of NPC cases in North America and Southern China, respectively, and is associated with Epstein-Barr virus (EBV) infection [1]. Global incidence shows a pattern associated with geographical location, where the highest prevalence is among the Chinese population in Southeast Asia and Southern China [2]. Although the involvement of genetic factors in NPC carcinogenesis is widely known, the mechanisms of their influence and/or action in the disease are not fully understood. This could be largely due to the fact that the full range of NPC-associated genes is still unclear. Among some of these are the ribosomal protein (RP) genes.

The products of RP genes canonically function as major components of ribosomes during protein biosynthesis. However, in 1996, Wool [3] listed more than 30 potential extraribosomal functions of RPs that include apoptosis, DNA repair, RNA processing, and transcription regulation. Sequence mutation and differential expression of several ribosomal protein genes have been reported in many human congenital disorders and carcinomas. For instance, more than 200 distinct mutations in nine RP genes, namely,* eS19 (S19), eS24 (S24), eS17 (S17), eS7 (S7), eS10 (S10), eS26 (S26), uL18 (L5), uL5 (L11)*, and* eL33 (L35a)*, were identified in a majority of Diamond-Blackfan Anemia (DBA) cases [4–10]. Genetic lesions in* eL22 (L22)* that include heterozygous deletion and nucleotide mutation have been found in T-cell acute lymphoblastic leukemia and colorectal cancer, respectively [11, 12]. Besides structural aberrancies, alteration in the expression pattern of RP genes has been found in many types of cancer. These include the dysregulated expressions of* uS3 (S3), eS19*, and* eS31* in colorectal cancer [13–15];* eL8 (L7a)*,* eL19 (L19)*,* eS27 (S27)*, and* eL37 (L37)* in prostate cancer [16–19];* eS27* in cells of breast tumor and gastric carcinoma; and* eL5* and* eL14 *in ovarian cancer [20–22]. Since 2014, a new naming system for RP genes has been introduced to unambiguously identify each RP gene across broad taxonomic range [23]. We adhere to this new system in this paper but have also included the old names (in parentheses) at first mention of the genes.

Association of RP genes with NPC was initially reported for* eS26 *and* eS27*. These two RP genes were downregulated in tumors of NPC relative to normal controls [24]. Three other genes (*eL27*,* eL41*, and* eL43*) were later proven to be NPC-associated RP factors in cell lines derived from NPC tissues [25, 26]. However, the hypothesis of* eS26* and* eS27* as NPC-associated RP genes was subsequently refuted [27]. Another RP gene,* eL32 (L32)*, was also found to be not involved with NPC tumorigenesis [27]. This means that only a limited subset of RP genes are associated with NPC. The complete list of genes that belong to this subset is yet to be resolved. Here, we provide evidence of four more RP genes that are relevant to the context of NPC tumorigenesis. They are identified on the basis of significant differential expression pattern between NPC and nonmalignant nasopharyngeal epithelial cell lines.

## 2. Materials and Methods

### 2.1. Cell Lines and Cell Culture

Six NPC-derived cell lines (HONE-1 [28], SUNE-1 [29], HK1 [30], TW01 [31], TW04 [31], and C666-1 [32]) and an immortalized nonmalignant nasopharyngeal epithelial cell line (NP69 [33]) were used in this study. Many of these were originally sourced from the University of Hong Kong (laboratory of Professor George S. W. Tsao) with permission for use granted by the provider. The NPC cell lines were cultured in RPMI-1640 (Gibco, Life Technologies, USA) containing 10% (v/v) fetal bovine serum, 2 mM L-glutamine, and 100 U/mL penicillin-streptomycin (Gibco, USA). The EBV-positive cell line, C666-1, was cultured on fibronectin-coated (Sigma, USA) cell culture flask containing prewarmed (37°C) RPMI-1640 medium. The NP69 cells were cultured in defined keratinocyte-serum-free medium (Invitrogen, USA) supplemented with 0.2 ng/mL growth factors, 5% heat-inactivated FBS, and 100 U/mL penicillin-streptomycin. All cells were maintained at 37°C in a humidified environment containing 5% CO_2_. Cells were harvested at a growth confluency of 70–80%.

### 2.2. Total RNA Extraction and Quantitative Reverse Transcription-PCR (qRT-PCR)

Total cellular RNA was extracted from the cell cultures using TRizol Reagent (Invitrogen, USA) according to the manufacturer's protocol. The extracted RNAs were DNase-treated with RQ1 RNase-Free DNase (Promega, USA). First strand cDNA was prepared using Moloney Murine Leukemia Virus Reverse Transcriptase (M-MLV RT; Promega, USA). Real-time PCR (or qPCR) was performed using Rotor-Gene™ SYBR Green PCR Kit (Qiagen, USA) and QuantiNova SYBR Green PCR Kit (Qiagen, USA) on a Rotor-Gene 6000 Rotary Analyzer (Qiagen, USA) and analyzed using Rotor-Gene 6000 software Version 2.3.3 (Qiagen, USA). For each assay, a total of 8 ng cDNA was added to a final reaction volume of 25 *μ*L containing 1x Rotor-Gene SYBR Green PCR master mix or QuantiNova SYBR Green PCR master mix and 1 *μ*M of each forward and reverse primer.  Table 1 lists the details of the PCR primers used for this study. Quantitative gene amplifications were performed using the following thermocycling conditions: initial denaturation for 5 minutes at 95°C, 40 cycles of denaturation at 95°C for 5 seconds, and annealing and extension at 60°C for 20 seconds. Glyceraldehyde-3-phosphate dehydrogenase* (GAPDH)* genes was used as endogenous control (or reference gene). Nontemplate reaction was used as negative control. Biological triplicate tests were done for all analysis.

The selection of the threshold intensity was set at a fixed intensity on the log-linear phase of the amplification curve for all the samples tested. Validation experiments, which included the generation of standard curves using a series of diluted cDNA samples, were carried out to ensure primer efficiency as well as target and reference gene amplification compatibility. Melt curve analysis and conventional agarose gel analysis were adopted alongside to verify the presence of a single amplicon. An interassay calibration scheme was adopted to minimize loading variation and to detect possible contamination with the inclusion of duplicate reactions and “no-template” control (NTC), respectively, in each qPCR assay. All samples were normalized to* GAPDH* as the endogenous control. Relative fold differences were calculated by the ΔΔC_T_ method [34].

### 2.3. Validation of PCR Efficiency

Primer efficiency was validated by generating a duplicate fivefold serial dilution over five-log magnitude. A calibration curve for each gene was plotted with average quantification cycle (*C*_*q*_) values against log input amount (4, 0.8, 0.16, 0.032, and 0.0064 ng/*μ*l). PCR amplification efficiency was determined from the slope of the log-linear portion of the calibration curve as follows:

(1) $$\begin{array}{c} Amplification\;\;efficiency=\left[\;10^{-1/slope}\;\right]-1. \end{array}$$

Valid ΔΔC_T_ calculation requires the PCR amplification efficiencies of the target and reference genes to be acceptably comparable. This was determined by evaluating the relative efficiencies of the target and reference amplification from the individually generated standard curves using the sample and log dilution. The Δ*C*_*q*_ values (C_T target_ − C_T reference_) were plotted against log input amount of five-template DNA dilution (4, 0.8, 0.16, 0.032, and 0.0064 ng/*μ*l). The slope of the resulting semi-log regression line (slope of Δ*C*_*q*_ versus log of input amount) was used to determine the compatibility of the two PCR efficiencies, with the slope value (*m*) less than 0.1 taken as ideal. In this study, primer efficiency of the respective genes in an NPC cell line (HK1) was validated against the reference gene,* GAPDH*. Quality assessment tests indicated that the efficiency curve for each of the primer sets relative to* GAPDH* was Correlation Coefficient (*R*^2^) greater than or equal to 0.97 and PCR efficiency (or amplification compatibility) of at least 90% (within the ideal amplification range of 90–110%). Correspondingly, the validation plot of ΔC_T_ versus log input amount of RNA shows *m* values within the range of −3.6 and −3.1, thus ascertaining the validity of our qPCR experiments.

### 2.4. Statistical Analysis

A mean fold difference value of more than 1.0 was considered to be an overexpression while a fold difference value of less than 1.0 was taken as an underexpression. The difference of means (target genes expression in NP69 and NPC cell lines) was statistically evaluated using unpaired Student's *t*-test, and statistical significance was taken at *p* < 0.05.

## 3. Results

### 3.1. Relative Expression Level of RP Genes in Each NPC Cell Line versus Normal Control

Fold difference of the 8 RP genes in each of the 6 NPC cell lines (HONE-1, SUNE-1, HK1, TW01, TW04, and C666-1) relative to the normal control (NP69) is shown in Table 2. Differential expression pattern can be as small as 0.03-fold (*uS4*; TW01 versus NP69) to as large as 131.92-fold (*eL18*; SUNE-1 versus NP69).* uS8*,* uS4*, and* uL14* are observed to be consistently underexpressed in all six NPC cell lines when compared to NP69, with the lowest expression observed in the SUNE-1 (0.07-fold), TW01 (0.03-fold), and SUNE-1 (0.17-fold) cell line, respectively. Consistent upregulation in expressions of* eL18* and* eL30* is observed in all six NPC cell lines relative to that of NP69, with the highest fold difference in SUNE-1 (133.92-fold) for* eL18*, and C666-1 (27.57-fold) for* eL30*. An interesting trend is observed in the expression levels of* eS31 *and* eL6* in which their general expression trend in NPC cell lines is inconsistent in HONE-1. For instance, expression of* eS31 *in NPC cell lines maintains an upregulated pattern except in HONE-1. A reversed scenario is evident for* eL6* where a downregulation trend is observed for all NPC cell lines except in HONE-1. Among the 8 RP genes tested, underexpression is statistically significant (*p* < 0.05) in all NPC cell lines relative to normal control for* uS8*,* uS4*, and* uL14*. In the case of* eS31*, significant downregulation is observed in all NPC cell lines except for HONE-1. Its upregulated pattern in HONE-1 is not statistically significant.

### 3.2. Expression Level of Each RP Gene Collectively in NPC Cell Lines Compared to NP69

**Figure 1:**
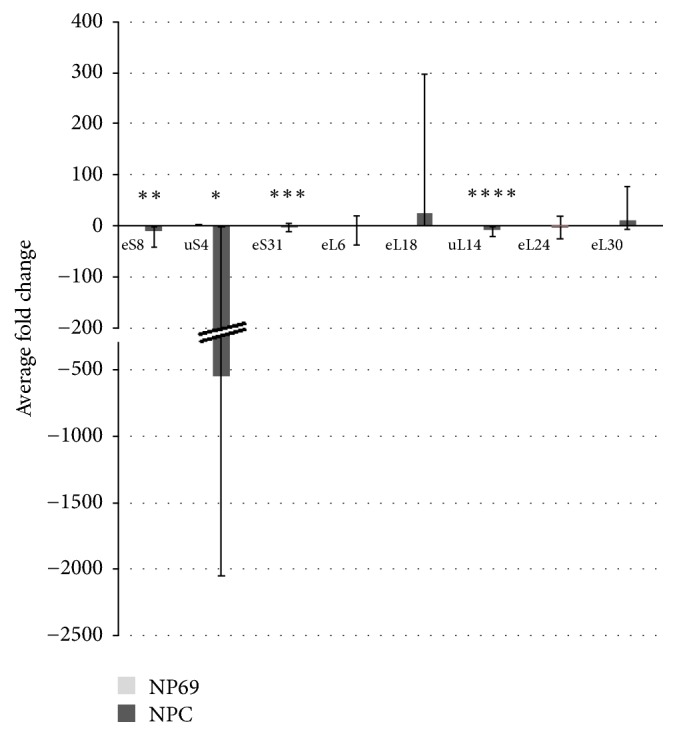
Relative fold difference of each RP gene in NPC cell lines versus normal control. The *y*- and *x*-axis represent average fold difference and ribosomal protein gene types, respectively. Data for each of the seven RP genes studied from in all the NPC cell lines studied were pooled and compared with data from the nonmalignant nasopharyngeal epithelial cell line, NP69. The *p* values of <0.05 were considered to be statistically significant and marked using asterisk symbols (^*∗*^*p* < 0.05, ^*∗∗*^*p* < 0.01, ^*∗∗∗*^*p* < 0.001, and ^*∗∗∗∗*^*p* < 0.00001). Error bars represent the upper and lower limits of the cumulative fold difference.

To examine the overall differential expression pattern of each RP gene in NPC, the mean fold difference of each gene in the six NPC cell lines was averaged and compared to that of the normal (nonmalignant) nasopharyngeal epithelial cell line, NP69 (Table 3, Figure 1). Underexpression pattern is observed for* eS8*,* uS4*,* eS31, uL14*, and* uL24*, with* uS4* displaying the highest underexpression level (−545.06-fold). However, only the downregulation pattern of* uL24 *is not statistically significant (*p* = 0.054). Overexpression trend is observed for* eL6, eL18*, and* eL30*. The expression of* eL18* recorded the highest upregulation of 25.64-fold. Nonetheless, these overexpression patterns are not statistically significant. All in all, significant differential expression can only be concluded for* eS8 *(*p* = 0.000166),* uS4 *(*p* = 0.023),* eS31 *(*p* = 0.00025), and* uL14* (*p* = 2.47^e−05^). These genes show an underexpression pattern in NPC cell lines compared to normal cell line.

## 4. Discussion

In summary, our studies reveal the significant downregulation of three 40S RP genes (*eS8, uS4*, and* eS31*) and a 60S RP gene* (uL14) *in NPC cell lines. Our findings reinforce previous reports [25, 26] on the occurrence of dysregulated expression among a subset of ribosomal protein genes in NPC. The differential expression pattern of* eS8, uS4*,* eS31*, and* uL14* is revealed for the first time in the NPC context and, hence, adds to the list of possible NPC-associated RP factors.

To date, differential expression of* eS8* gene and its protein has been found in colorectal tumors/polyps [13] and colorectal carcinoma [35], respectively. However, its expression is constitutive and ubiquitous among normal and neoplastic thyroid tissues and cell lines [36–38]. This inconsistency of* eS8*'s expression characteristics across different types of cancer suggests its distinctive association with the type of malignant tissue/cell. More studies will be required to substantiate this. Molecularly, eS8 protein interacts with Cyclin Dependent Kinase 11B (CDK11B), a key mediator of Fas ligand-induced apoptosis [39]. It remains to be investigated whether downregulation of* eS8* affects a disrupted eS8-CDK11B interaction and, thus, is associated with dysregulated cellular apoptotic pathway in NPC cells.

Differential expression of* uS4* in colorectal cancer [40] is not consistently observed for human lung squamous cell, oral squamous cell, and liver cancer [41–43]. Similar to* eS8*, the association of* uS4* with cancers is likely to be type-specific. Silencing of* uS4* in glioma cells affects morphological differentiation without causing senescence [44]. In human embryonal carcinoma NTERA2 cells,* uS4 *is consistently underexpressed during retinoic acid induced neuronal differentiation [45]. It seems that there is relationship between the downregulation of* uS4* and cellular differentiation. Our findings here corroborate with this notion. The level (number of fold differences) of* uS4 *underexpression in TW04 and C666-1 cell lines is the lowest, followed by HONE-1 and SUNE-1, and the highest in HK1 and TWO1 (Table 2). TWO4 and C666-1 are undifferentiated cell lines, while HONE-1 and SUNE-1 are from poorly differentiated squamous cell carcinoma, and HK1 and TW01 are from differentiated squamous carcinoma. The connection between* uS4* expression and the degree of cellular differentiation warrants more research in order to be fully understood. At this stage, it implies that this RP can be a possible differentiation marker amenable for accurate histopathological analysis of NPC cells/tissues.

The eS31 is an ubiquitin C-terminal extension protein that is overexpressed in colorectal [15], breast [46], and renal [47] cancers and leukemia [48]. It is, however, ubiquitous in gastric and ovarian cancers [49] and downregulated in hepatocellular carcinoma (HCC) tissues [50]. During ribosomal stress,* eS31 *is overexpressed and the translated product mediates activation and stabilization of p53 by binding with MDM2 protein to inhibit MDM2-mediated p53 ubiquitination [51, 52]. Correspondingly, knockdown of* eS31* mitigates p53 activation in response to ribosomal stress [52]. This implies a positive correlation between expressions of* eS31 *and* p53*. However, studies have shown that* p53* is overexpressed and largely unmutated in NPC tissues and cells [53–55]. Our findings of* eS31*'s underexpression in NPC cells also appear unrelated to the trend of* p53*'s expression pattern in this type of cancer. This confusion is difficult to understand without deeper studies into the relationship between* eS31 *and* p53 *during malignant condition of NPC cells. Nevertheless, it does suggest the notion that eS31 possibly fails to regulate p53 in the cancer situation.

The expression of* uL14* is upregulated in squamous cell carcinoma of the head and neck [56]. On the other hand, it is underexpressed in ovarian cancer [22] and human papillomavirus-16 E6 D25E-expressing cell lines [57]. Increase in uL14 protein inhibits HDM2-induced p53 ubiquitination [58] and would contribute to activation and increase of p53. The knockdown of* uL14* affects actinomycin D-induced p53 activation [59]. These facts imply a positive correlation between uL14 and p53 levels. However, in a study by Fumagalli et al. [60], the perturbation of either 40S or 60S ribosomal biogenesis, which included the depletion of uL14, coincides with the induction of p53 in A549 human lung carcinoma and U2OS human osteosarcoma cell lines. This is consistent with our findings and reinforces the notion of negative correlation between* uL14* expression and p53 levels in some cancer types, in our case, NPC. The molecular explanation to this would require further research.

Besides the cancer-type specificity of expression level of the 4 newly identified NPC-associated RP genes, this study has also revealed another 4 RP genes (*eL6, eL18*,* eL24*, and* eL30*) that are not relevant to the context of NPC tumorigenesis. This underpins our suspicion of the limited selection of RP genes that are linked to the NPC disease. The network of their activities in the carcinogenesis of the nasopharynx will be a potent area for future studies.

## 5. Conclusion

The ribosomal protein genes of* eS8*,* uS4*,* eS31*, and* uL14* are significantly underexpressed in NPC cell lines relative to nonmalignant nasopharyngeal epithelial cells. These genes represent the latest addition to the limited list of RP genes that have association with cancer of the nasopharynx.

## Figures and Tables

**Table 1:** Target genes and the corresponding primer sequences, product size, and correlation coefficient of efficiency curve. F and R represent the forward and reverse primer, respectively.

Gene name	GenBank accession number	Primer sequence (5′-3′)	Expected product size (bp)
*GAPDH*	NM_002046.5	F: CTGGGCTACACTGAGCACC	101
		R: AAGTGGTCGTTGAGGGCAATG	
*uS8*	NM_001012.1	F: GCTCAGAGTGTTGTACTCG	106
		R: AGCACGATGCAATTCTTCAC	
*uS9*	NM_001013.3	F: CTGAAGTTGATCGGCGAGTATG	280
		R: ACTTGGCCAAGCCCAGCTTG	
*eS31*	NM_002954.5	F: GCAGCTGGAAGATGGACGTAC	85
		R: ACCACCACGAAGTCTCAAC	
*eL6*	NM_001024662.1	F: GCACGTGAGAACACTGCGAG	139
		R: GAGGACCCGAGCTCCAGTCAC	
*eL18*	NM_000979.3	F: CTCTGTCCCTTTCCCGGATG	320
		R: GTAGGGTTTGGTGTGGCTG	
*uL14*	NM_000978.3	F: TCCAGCAGTGGTCATTCGAC	117
		R: GCAGAACCTTTCATCTCGCC	
*eL24*	NM_000986.3	F: CGAGCTGTGCAGTATTAGCG	117
		R: GAAAGGAAAGCCGACTCGC	
*eL30*	NM_000989.3	F: ATCTTAGTGGCTGCTGTTGG	280
		R: TGCCACTGTACTGATGGACAC	

**Table 2:** Fold difference of the studied RP genes in NPC cell lines relative to normal control. Data in this table includes normalized mean fold difference (2^−ΔΔ*C*_*q*_^) of respective genes in six NPC cell lines relative to normal nasopharyngeal epithelial cell line, NP69.

Gene	Cell line	Fold difference (2^−ΔΔ*C*_*q*_^)	Std. deviation (SD)	*p* value
*eS8*	NP69	1.012746	0.011882	
	HONE-1	0.380311	0.273122	0.008019
	SUNE-1	0.071494	0.009687	2.34^*e*−08^
	HK1	0.251844	0.163468	0.000649
	TWO1	0.402658	0.44444	0.038129
	TWO4	0.253709	0.282225	0.004816
	C666-1	0.196327	0.085366	4.04E-05

*uS4*	NP69	1.21591	0.314452	
	HONE-1	0.164457	0.137157	0.003026
	SUNE-1	0.203615	0.345601	0.009952
	HK1	0.149012	0.225628	0.004405
	TWO1	0.032517	0.028321	0.001452
	TWO4	0.416219	0.29763	0.016465
	C666-1	0.216689	0.22373	0.005476

*eS31*	NP69	1.013452	0.010872	
	HONE-1	2.637661	2.117031	0.127319
	SUNE-1	0.182123	0.068557	1.6^*e*−05^
	HK1	0.560767	0.239252	0.015339
	TWO1	0.415694	0.235145	0.005853
	TWO4	0.246348	0.160656	0.000588
	C666-1	0.322675	0.329258	0.011062

*eL6*	NP69	1.080437	0.043553	
	HONE-1	0.298751	0.235938	0.002428
	SUNE-1	5.478057	5.216626	0.109026
	HK1	10.04366	8.672146	0.073961
	TWO1	2.92429	1.745115	0.070656
	TWO4	6.398342	4.511504	0.055374
	C666-1	2.365366	0.344513	0.001523

*eL18*	NP69	1.02048	0.005098	
	HONE-1	2.72834	1.206147	0.035128
	SUNE-1	131.9168	149.1636	0.101579
	HK1	3.345807	2.137831	0.066337
	TWO1	1.467657	0.354116	0.047003
	TWO4	1.900801	0.691558	0.046081
	C666-1	12.5088	6.650983	0.020133

*uL14*	NP69	1.123026	0.152195	
	HONE-1	0.374748	0.294766	0.00872
	SUNE-1	0.172974	0.057157	0.000268
	HK1	0.305362	0.199669	0.002431
	TWO1	0.189579	0.093481	0.000413
	TWO4	0.308219	0.190789	0.002221
	C666-1	0.177184	0.184382	0.001187

*eL24*	NP69	1.058277	0.07439	
	HONE-1	8.01882	9.602711	0.138828
	SUNE-1	0.426845	0.363781	0.02108
	HK1	1.807239	1.8652	0.262673
	TWO1	0.504738	0.403963	0.039945
	TWO4	0.573587	0.461182	0.073365
	C666-1	0.191813	0.13318	0.000299

*eL30*	NP69	1.072429	0.06969	
	HONE-1	5.27733	4.672817	0.097009
	SUNE-1	2.217279	3.501794	0.300566
	HK1	21.05978	31.37735	0.165887
	TWO1	8.75249	10.80404	0.142807
	TWO4	5.414118	7.185418	0.177129
	C666-1	27.56961	43.37632	0.174837

**Table 3:** Relative expression of each RP gene in NPC cell lines compared to NP69. ^*∗*^Fold difference values <1.0 were converted to linear-scale with the formula linear FD = (−1/FD).

Gene	Cell line	Mean linear FD^*∗*^	*p* value	Expression pattern
*eS8*	NP69	1.012746	0.000166	Significant underexpression
	NPC	−9.32115		
*uS4*	NP69	1.21591	0.023017	Significant underexpression
	NPC	−545.064		
*eS31*	NP69	1.013452	0.00025	Significant underexpression
	NPC	−3.34067		
*eL6*	NP69	1.080437	0.691146	Overexpression
	NPC	2.133808		
*eL18*	NP69	1.02048	0.07951	Overexpression
	NPC	25.6447		
*uL14*	NP69	1.123026	2.47^*e*−05^	Significant underexpression
	NPC	−7.43259		
*eL24*	NP69	1.058277	0.053756	Underexpression
	NPC	−3.74191		
*eL30*	NP69	1.072429	0.075329	Overexpression
	NPC	10.82959		
